# Artificial intelligence for improving Nitrogen Dioxide forecasting of Abu Dhabi environment agency ground-based stations

**DOI:** 10.1186/s40537-023-00754-z

**Published:** 2023-06-02

**Authors:** Aamna AlShehhi, Roy Welsch

**Affiliations:** 1 Biomedical Engineering, Khalifa University, Abu Dhabi, United Arab Emirates; 2 Sloan School of Management and Statistics, Massachusetts Institute of Technology, Cambridge, Massachusetts USA

**Keywords:** Nitrogen Dioxide, Forecast, Artificial Intelligence, Deep Learning, Temporal Models, Transformer Model

## Abstract

Nitrogen Dioxide (NO$_{2}$) is a common air pollutant associated with several adverse health problems such as pediatric asthma, cardiovascular mortality,and respiratory mortality. Due to the urgent society’s need to reduce pollutant concentration, several scientific efforts have been allocated to understand pollutant patterns and predict pollutants’ future concentrations using machine learning and deep learning techniques. The latter techniques have recently gained much attention due it’s capability to tackle complex and challenging problems in computer vision, natural language processing, etc. In the NO$_{2}$ context, there is still a research gap in adopting those advanced methods to predict the concentration of pollutants. This study fills in the gap by comparing the performance of several state-of-the-art artificial intelligence models that haven’t been adopted in this context yet. The models were trained using time series cross-validation on a rolling base and tested across different periods using NO$_{2}$ data from 20 monitoring ground-based stations collected by Environment Agency- Abu Dhabi, United Arab Emirates. Using the seasonal Mann-Kendall trend test and Sen’s slope estimator, we further explored and investigated the pollutants trends across the different stations. This study is the first comprehensive study that reported the temporal characteristic of NO$_{2}$ across seven environmental assessment points and compared the performance of the state-of-the-art deep learning models for predicting the pollutants’ future concentration. Our results reveal a difference in the pollutants concentrations level due to the geographic location of the different stations, with a statistically significant decrease in the NO$_{2}$ annual trend for the majority of the stations. Overall, NO$_{2}$ concentrations exhibit a similar daily and weekly pattern across the different stations, with an increase in the pollutants level during the early morning and the first working day. Comparing the state-of-the-art model performance transformer model demonstrate the superiority of ( MAE:0.04 (± 0.04),MSE:0.06 (± 0.04), RMSE:0.001 (± 0.01), R$^{2}$: 0.98 (± 0.05)), compared with LSTM (MAE:0.26 (± 0.19), MSE:0.31 (± 0.21), RMSE:0.14 (± 0.17), R$^{2}$: 0.56 (± 0.33)), InceptionTime (MAE: 0.19 (± 0.18), MSE: 0.22 (± 0.18), RMSE:0.08 (± 0.13), R$^{2}$:0.38 (± 1.35) ), ResNet (MAE:0.24 (± 0.16), MSE:0.28 (± 0.16), RMSE:0.11 (± 0.12), R$^{2}$:0.35 (± 1.19) ), XceptionTime (MAE:0.7 (± 0.55), MSE:0.79 (± 0.54), RMSE:0.91 (± 1.06), R$^{2}$: $-$4.83 (± 9.38) ), and MiniRocket (MAE:0.21 (± 0.07), MSE:0.26 (± 0.08), RMSE:0.07 (± 0.04), R$^{2}$: 0.65 (± 0.28) ) to tackle this challenge. The transformer model is a powerful model for improving the accurate forecast of the NO$_{2}$ levels and could strengthen the current monitoring system to control and manage the air quality in the region.

**Supplementary Information:**

The online version contains supplementary material available at 10.1186/s40537-023-00754-z.

## Introduction

Humanity faces many global environmental challenges embedded in global warming, environmental degradation, biodiversity loss, and poor air quality [1–3]. Poor air quality, which contains a high level of gaseous air pollutants, negatively impacts human health by causing respiratory and pulmonary diseases and the environment by contributing to climate change and acid rain [1, 3–9]. The deterioration in the air quality was associated with rapid social development and urbanization, which increased human activities such as vehicle usage, traffic, cooking, and building cooling and heating [4]. In addition to the air pollutants produced by human activities, pollutants are also released from nature [1]. One of the major air pollutants is Nitrogen dioxide (NO$_{2}$).

NO$_{2}$ is a toxic pollutant made up of nitrogen and oxygen atoms [10].The pollutant level increase in the air due to human and natural sources such as vehicles, aviation, manufacturing, power plants, indoor pollutant, soil processes, and lightning [3, 7–12].It is also known NO$_{2}$ plays a vital role in increasing the density of other hazardous outdoor air pollutants such as ground-level ozone (O$_{3}$) and fine particles (PM$_{2.5}$) [11–13]. In 2019, NO$_{2}$ was estimated to cause 637,000 new pediatric asthma incidents in China [7] and 1.85 million new cases globally [11].Besides asthma, it increases the risk of other diseases, such as cardiovascular mortality, respiratory mortality, and lung cancer incidence or mortality [3, 13]. To mitigate the pollutant’s adverse effects, the World Health Organization (WHO) issued new Air Quality Guidelines (AQG) on September 22, 2021, to set the annual average threshold of NO$_{2}$ concentration to 10 micrograms per cubic ($\mu$g/m$^{3}$) [7]. The scientific community also supported the efforts by conducting several epidemiological studies to reveal the association between pollutants and diseases and computational analyses to understand the pollutants’ pattern and predict pollutants’ future concentration. The previous computational analysis studies supported the atmospheric management decision-makers to track and monitor the pollutant level and issue applicable regulations and laws to reduce the adverse risk of NO$_{2}$ pollutants (for sure by understanding its causes). These early warning system projects are directed toward understanding the pollutant’s hourly, daily, monthly, and annual concentration pattern [14], investigating the impact of different unexpected interventions such as the COVID-19 pandemic on it is level [11, 12, 15], and predict future pollutant concentration using statistical, machine learning (ML), and artificial intelligence (A.I.) methods [3, 8, 9]. The latter methods have recently gained much attention due it’s capability to tackle complex and challenging problems in computer vision, natural language processing, etc. Air quality prediction is also categorized as challenging and complex tasks that faces humanity due to the fact that the pollutants concentration is correlated and associated with several environmental and physical factors such as meteorological, traffic pollution, and industrial emissions that vary across time and space [8, 16]. Surveying the previous scientific efforts to build predictive models, earlies efforts focused in using classical statistical methods such as the auto-regressive model (AR), moving aver- age model (MA), auto-regressive integrated moving average model (ARIMA), and seasonal ARIMA (SARIMA) [17]. While the recent works moved toward utilized ML and A.I. algorithms such as the multilayer perceptron model (MLP) [1], long short-term memory (LSTM) [8],and Bidirectional convolutional LSTM [3]. The shift towards ML and A.I. algorithms, which remarkably outperform the performance of classical statistical methods, improves pollutant forecasting since those methods automatically learn and extract the features from the data and use the new data representation (extracted features) for generalization to the unseen data [8].

Even though the recent studies provide clear evidence of the power of ML and A.I. to improve the prediction of the future NO$_{2}$ concentration with best reported R$^{2}$ range from 0.87 to 0.9 and RMSE range from 0.21 to 19.14, the domain is still in its infancy to tackle this challenge. In the NO$_{2}$ context, there is still a research gap in adopting those advanced deep learning for sequences data to predict the concentration of pollutants, as an example of unadopted techniques, Transformer for time series, MINImally RandOm Convolutional KErnel Transform (MiniRocket), InceptionTime etc.

The main objective of this study is to explore the NO$_{2}$ temporal characteristics along with comparing and validating the performance of several state-of-the-art A.I. models, namely: MINImally RandOm Convolutional KErnel Transform (MiniRocket) [18], Residual Network (ResNet) for time series [19], XceptionTime [20], InceptionTime [21] and Transformer for time series [22] to improve the accuracy of NO$_{2}$ forecasting. We trained our models using data collected and provided by Abu-Dhabi. Environment Agency- Abu Dhabi (EAD), United Arab Emirates (UAE), for different environmental monitoring stations. To recapitulate, the contributions of the paper are as follows:

- This is the first study that investigates the temporal characteristics of NO$_{2}$ concentration across 19 stations covering seven environmental assessment points in the UAE.
- This work is among the first comprehensive work to adopt and compare the performance of several state-of-the-art deep learning models to improve the accuracy of forecasting future NO$_{2}$ concentration.

## Methods

### Study area

UAE was established in 1971 and consists of seven emirates: Abu Dhabi, Dubai, Sharjah, Ajman, Umm Al Quwain, Ras Al Khaimah, and Fujairah. Abu-Dhabi, the UAE’s capital and the largest emirate accounts for 87% (67,000 km$^{2}$) of the total area with 23.5$^{\circ }$N 54.5$^{\circ }$E geographic coordinates [15].

### In-Situ observation data of NO${_2}$ concentration

**Fig. 1:**
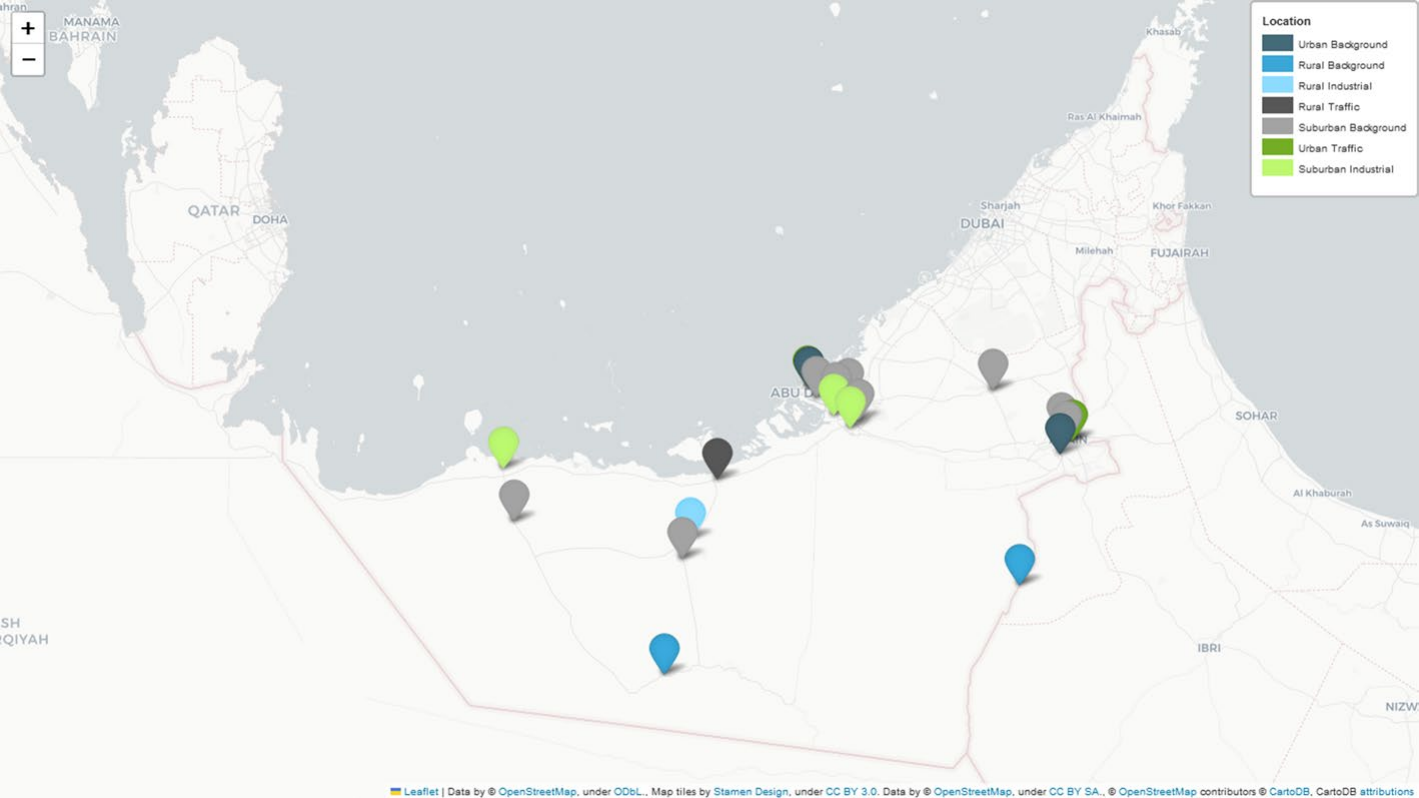
The study area map The geographic distribution of the 20 NO$_{2}$ stations across UAE

This study focuses on NO$_{2}$ concentration prediction for several air quality stations in Abu Dhabi, which were collected and provided by the EAD. EAD is the environment regulator that aims to protect and enhance the region’s air quality, groundwater, and biodiversity. Since 2007, the agency started to collect and monitor air quality data; by operating 20 fixed ground stations with annual data capture of air quality is approximately 75% [23] in addition to 2 mobile stations across three regions in Abu Dhabi: Al Ain Region (Eastern Region), Al Dhafra Region (Western Region), and Central Region (Greater Abu Dhabi and it’s surrounding) (Fig. 1). The stations cover seven environmental assessment points: urban traffic, urban background, rural traffic, rural background, rural industrial, suburban background, and suburban industrial. The monitoring stations provided with air quality and meteorological sensors to record wind speed, wind direction, temperature, relative humidity, net radiation, barometric pressure, and pollutants such as Sulfur Dioxide (SO$_{2}$), Nitrogen Dioxide (NO$_{2}$), Ozone (O$_{3}$), Carbon Monoxide (CO), particulate matter (PM) and Hydrogen Sulfide (H2S) [24–26]. The monitors follow the technical testing standards of ISO/IEC 17,025:2017. The pollutants data measured across all the stations were transmitted to the Air Quality Management System database. The dataset gets further quality inspection, control, assessment, verification, and statistical processing to be presented on the EAD web portal (https://www.adairquality.ae). All the air pollution measurement systems follow ISO, CEN/EN, and U.S. standards [15]. Our focus in this study is NO$_{2}$ micrograms per cubic ($\mu$g/m$^{3}$) concentration data collected from 20 fixed ground stations from January 1, 2019, at 0:00 to December 31, 2020, at 23:00. For each station, we provided with 17,544 hly NO$_{2}$ concentration values.

### Data Pre-processing

**Fig. 2:**
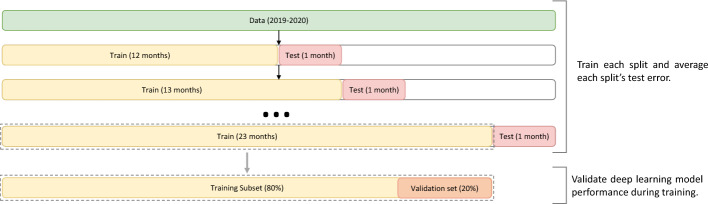
Time Series Cross-validation: The training set increases sequentially, maintaining the temporal order of the data for predicting one month ahead (testing set). The training set divided further into a training set and validation set using 80:20% for monitoring the models performance during training

We used rolling k-fold cross-validation for training different models. We divide each station’s data into training and testing sets as in (Fig. 2). In the begin the training set -which we used to train different deep learning models- consists of the historical data from January 1, 2019, 0:00 until December 31, 2019, 23:00 (12 months) while the testing set -which we will use to evaluate and compare the performance of different models- consists of the data from January 1, 2020 0:00 until January 31, 2020 (one month ahead). We repeated the process in which every-time we add one more month to the training set and used them to predict one month ahead.

Our data contains missing values which is expected from a real-life data (Additional file 1: Table S1 and Figures S1–S19); we notice a high percentage (62.64%) of NO$_{2}$ concentrations values were missing from station 13; therefore, we excluded this station form the future analysis. To deal with missing values for the remaining stations, we applied univariate time series weighted moving average; this technique outperforms other techniques for dealing with time series missing values, as reported in [27]. Precisely for this study, the exponential weighted moving average (EWMA) technique with five moving average windows was applied:

1 $$\begin{aligned} EWMA_{t} = w*Z_{t} + (1-w)*Z_{t-1} \end{aligned}$$

Where, Z$_{t}$ is the value of the series at time *t*; *EWMA*$_{t}$ is the EWMA value at time *t*. *w* represents the weighting factors that decrease exponentially, e.g., *at time (t-x,t+x)*; *w*= $\frac{1}{2^x}$; where x is observations directly next to a central value. After imputing the missing values, we average the daily NO$_{2}$ concentration for each station and use the averaged values in this work for further analysis. Before training the different models, the input data was standardized by removing the mean and scaling to unit variance. We further divide our training data into two data sets: 80% training set and 20% validation set (Fig. 2); the validation set used to monitor and prevent overfitting during the training of different deep learning models by comparing validation errors to the training error over epochs.

### Temporal characteristics of NO$_{2}$ pollutant emissions

To reveal the annual trend of the univariate NO$_{2}$ daily level, we used the seasonal Kendall test [14, 28]. It is a nonparametric test for testing the time series’ monotonic or consistent upward or download trends. The Seasonal Kendall S$_{k}$ statistic is computed as following:

2 $$\begin{aligned} S_{k} = \sum _{i=1}^{m} S_{i} \end{aligned}$$

Where *m* is the total number of seasons, and S$_{i}$ is *ith* season *S* from *m*. S$_{i}$ is Mann-Kendall, which is computed using the following equation:

3 $$\begin{aligned} S= \sum _{i=1}^{n-1} \sum _{j=i+1}^{n} sign(y_{j}-y_{i}) \end{aligned}$$

Where *S* computes the difference between the future measure values y$_{j}$ and all the previous values y$_{i}$. The *sign(y*$_{j}$-y$_{i}$) is +1 (positive differences),0 (no differences), or -1 (negative differences).

After computing and summing the seasonal statistics (S$_{k}$), the normalized Z$_{sk}$ test statistic is computed as follows:

4 $$Z_{{sk}} = \left\{ {\begin{array}{*{20}l} {\frac{{S_{k} - 1}}{{\sqrt {Var(S_{k} )} }}} \hfill & {{\text{ if}}\;S_{k} > 0.} \hfill & {} \hfill \\ 0 \hfill & {{\text{ if}}\;S_{k} = 0.} \hfill & {} \hfill \\ {\frac{{S_{k} + 1}}{{\sqrt {Var(S_{k} )} }}} \hfill & {{\text{ if}}\;S_{k} > 0.} \hfill & {} \hfill \\ \end{array} } \right.$$

The positive value of the normalized Z$_{sk}$ imply an increased trend in the series, and the negative values indicate a decreased trend.

We also computed Theil-Sen’s Slope Estimator [28, 29], a nonparametric method used to quantify the change in the time series magnitude: direction and volume. This technique is robust since it is not affected by outliers present. The slope of two points in the time series is computed using the following equation:

5 $$\begin{aligned} Q = \frac{x_{i}-x_{j}}{i-j} k \ne j \end{aligned}$$

Where *i* and *j* are two points in the time series. Sen’s method estimated slope (*Q*$^{*}$) as the median N values of Q; the *Q*$^{*}$ estimated as following:

6 $$Q^{*} = \left\{ {\begin{array}{*{20}l} {Q_{{(n + 1)/2}} } \hfill & {{\text{if }}N\,{\text{is odd}}} \hfill \\ {\frac{{Q_{{N - 2}} + Q_{{(N + 2)/2}} }}{2}} \hfill & {{\text{if }}N{\text{ is even}}} \hfill \\ \end{array} } \right.$$

7 $$N = {\text{ }}\frac{{n(n - 1)}}{2}{\text{ }}$$

Where *n* is the total number of samples in the time series, all the statistical analyses were tested at the 95% significance level with a two-tailed test.

### Predictive deep learning models

In this study, the daily NO$_{2}$ concentration was predicted using several state-of-the-art deep learning models for time series and sequences, namely: MINImally RandOm Convolutional KErnel Transform (MiniRocket) [18, 30], Residual Network (ResNet) for time series [19], XceptionTime [20], InceptionTime [21] and Transformer for time series [22].

- MiniRocket: is a high-speed, lesser computational state-of-the-art deep learning model. The methods select 10,000 non-random kernels with size 9 to generate model feature maps. Those kernels will vary in terms of the padding, dilation, non-trainable weights, and non-trainable bias. The model uses those fixed, non-trainable, and independent random convolutional kernels to extract a new feature (features maps) from the input sequence. The generated feature maps are fed to the proportion of positive values (PPV) pooling which used to detect a specific patterns from the input. Finally, it will pass into a linear model such as the ridge regression model or deep learning head for prediction.
- ResNet:is a deep learning model consisting of three residual blocks with linear residual connection to reduce the vanishing gradient effect exhibited due to the increase of the network depth followed by a 1D global Average pooling layer. Each residual block consists of three convolutions layers with 7, 5, and 3 kernel filters followed by a 1D convolution layer; it ends with a batch normalization layer and Rectified Linear Unit (ReLU) activation function.
- XceptionTime: architecture consists of stacking several XceptionTime modules with residual connection (a 1X1 Conv layer and batch normalization). In which the ReLU activation function is applied to the residual connection and the XceptionTime module feature map to introduce non-linearity in the network. The modules are followed by an adaptive average pooling layer to reduce overfitting and increase the robustness of the network to learn the temporal translation of the input sequence, and finally, several 1X1 convolution layers with batch normalization and ReLU. XceptionTime module includes two parallel paths: the first path has a 1X1 convolution layer followed by three Depthwise Separable Convolutions with different/multiple one-dimensional kernels to extract long and short-time dependency series features simultaneously. At the same time, the second path has a max pooling layer followed by a 1X1 Convolution layer. The module output consists of concatenating the feature maps learned by the two paths.
- InceptionTime: The network consists of two residual blocks: each with three inception modules and two linear skip-connection (1X1 convolution layer), followed by global average pooling. The inception module contains two parallel paths: the first path has a bottleneck layer (one-dimensional Convolutional Neural Network (1DCNN)) that works as a dimensionality reduction to reduce the number of parameters and improve model generalization; the 1DCNN is followed by three parallel depthwise separable convolutions and pointwise convolutions layers with different filter sizes to learn long and short time dependency features. The second path has one MaxPooling followed by a bottleneck layer. The output of the inception module consists of the concatenation of the feature maps generated by two paths. Also, in this network, ReLU is used as an activation function. Similar to the XceptionTime, this model also adopts the one-dimensional: convolutional, max pooling, and batch normalization to apply for temporal data. The final network consists of ensembling five different inception networks with different weights and initialization to improve network stability.
- Transformer or Transformer-decoder architecture: The model learns the long-term dependency in the sequence using a self-attention mechanism that gives more attention to the important subsets of the sequence over unimportant set. The model core component is the encoder part of the original transformer network to learn a new representation for the time series. The model needs to learn the association between previous tokens for encoding the current token. Each of the tokens will be assigned with query, key, and value. The query and the key will be used to decide the relationship between the current token and the previous one. While the value defines the new representation of the current token. The self-attention score of the previous and current tokens is calculated as the dot product of keys with queries, which will be fed to a softmax layer and scaled to create a ’soften’ probability distribution. The highest attentions score indicates a higher relevance between the current token and the previous token and vice versa. Finally, the current token’s encoder is calculated using the dot product of the scaled attention scores and token value vector. To account for the temporal characteristic of the time series, the positional encoding is added to the calculate the relative distance between the current token and the previous one. Since the input has a temporal resolution, the network used 1-DCNN to compute the keys and queries of the self-attention layer and positional encoding. Moreover, the model replaces layer normalization with batch normalization after the self-attention layer to alleviate the outliers’ issue in the time series dataset.

Table 1 presents the hyperparameters used to train the different deep learning models. All the models were trained using 100 epochs with 64 batch sizes,sequence of length 10,and Adam optimizer. In this study, we used a fixed architecture component for each mode; as reported in Table 1, we only tuned the learning rate for each station and model pair. The suggested learning rate was selected based on the valley algorithm.

**Table 1:** Deep learning models selected hyperparameters used during training

Model	Hyperparameters
MiniRocket	Number of features: 10000; Maximum dilations per kernel: 16; scoring: MSE
ResNet	Windows size = 24, filter size = 32, kernel sizes: 7, 5 and 4
XceptionTime	Filter size = 16, adaptive average pooling: 32
InceptionTime	Filter size = 32, kernel sizes: 24, depth : 6; dilation: 1
Transformer	Windows size = 24, embedding size: 32, Size of the intermediate
	feed forward layer:16, number of layers: 2 and number of heads: 4

### Benchmarking

We benchmark our study using Long Short-Term Memory (LSTM) model to compare the performance of the state-of-the-art models against. LSTM is a recurrent neural network (RNN) for analyzing sequence data. It addresses long-term dependency problem which cause vanishing gradient problem in the RNN model. LSTM introduces three gates: forget gate, input gate, and output gate; those gates control the network memorizing process: read, store, and write historical information [31].

### Models performance evaluation metrics

Four evaluation measures are used to evaluate and compare the performance of the different models, precisely, correlation coefficient (R$^{2}$), mean square error (MSE), root means square error (RMSE), and mean absolute error (MAE) [1].

The analyses were performed using R programming language (version 3.6.1): imputeTS [32] package (version 3.2) to impute time series missing values. In addition to several Python (version 3.8.13) packages: tsai [33](version 0.3.1) to train the deep learning models, scikit-learn (version 1.1.1) to compute the evaluation metrics, and pymannkendall ( version 1.4.2)to calculate the temporal characteristics of the time series.

## Results

### Temporal characteristics of NO$_{2}$ pollutant emissions

**Fig. 3:**
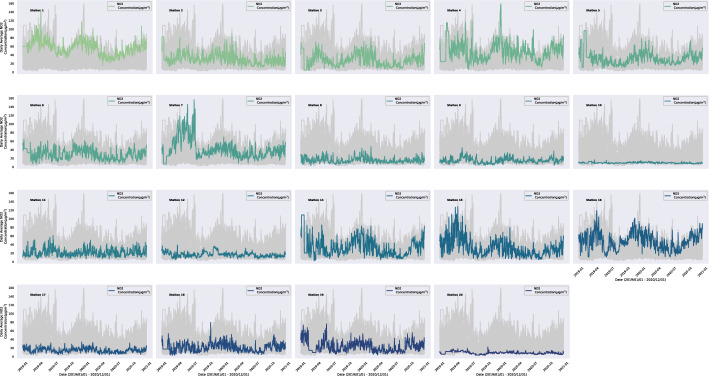
NO$_{2}$ concentration Daily mean NO$_{2}$ concentration of 19 stations during the period from 1/1/2019–31/12/2022. The gray curves represent all the stations’ curves, while the colored curve represents the specific station trend

The geographical study area of this work is the UAE; specifically, its capital Abu Dhabi. Figure 3 shows the average daily NO$_{2}$ concentration for the 19 monitoring stations from January 1, 2019, to December 31, 2020; in parallel, table 2 presents the statistical description of the NO$_{2}$ concentration for each station and trend statistical test. The monitoring stations cover seven environmental assessment points: urban traffic, urban background, rural traffic, rural background, rural industrial, suburban background, and suburban industrial.

**Table 2:** Statistical descriptions of the NO$_{2}$ concentration for the 19 stations (Unit: micrograms per cubic ($\mu$g/m$^{3}$))

Station	Mean	Std.	Min	Max	Mann-Kendall	P-value	Theil-Sen’s	Trends
					seasonal statistical test		Slope Estimator	
Station 1	56.44	17.09	15.36	143.03	−179	0.0 *	−10.51	Decreasing
Station 2	31.42	13.97	7.40	89.71	−165	0.0 *	−9.70	Decreasing
Station 3	27.89	14.86	3.92	80.07	−85	0.0 *	−5.17	Decreasing
Station 4	47.8	21.98	5.74	159.70	−11	0.6 *	−0.83	No trend
Station 5	32.31	15.56	8.85	96.67	55	0.00 *	2.51	Increasing
Station 6	29.7	12.17	6.23	64.51	−111	0.00 *	−5.19	Decreasing
Station 7	42.13	23.69	7.156	156.62	−127	0.00 *	−9.35	Decreasing
Station 8	15.73	6.72	3.44	47.79	−19	0.35 *	−0.74	No trend
Station 9	14.85	6.23	3.95	45.00	61	0.00 *	1.60	Increasing
Station 10	9.63	1.44	6.60	16.86	−60	0.00 *	−0.32	Decreasing
Station 11	21.68	9.01	6.64	61.11	15	0.47 *	0.41	No trend
Station 12	16.68	5.75	5.08	39.5	−113	0.00 *	−2.73	Decreasing
Station 14	33.79	21.05	2.36	109.13	−135	0.00 *	−7.50	Decreasing
Station 15	32.70	20.69	4.58	128.26	−181	0.0 *	−12.15	Decreasing
Station 16	47.86	19.68	11.2	119.31	−51	0.01 *	−3.40	Decreasing
Station 17	14.81	5.44	4.02	33.90	−103	0.00 *	−2.66	Decreasing
Station 18	21.27	10.02	2.97	79.31	−75	0.00 *	−2.88	Decreasing
Station 19	23.38	12.77	3.51	76.33	−71	0.00 *	−4.76	Decreasing
Station 20	8.60	2.94	2.77	22.53	−71	0.00 *	−1.18	Decreasing

$$^{*}$$p<.05

**Fig. 4:**
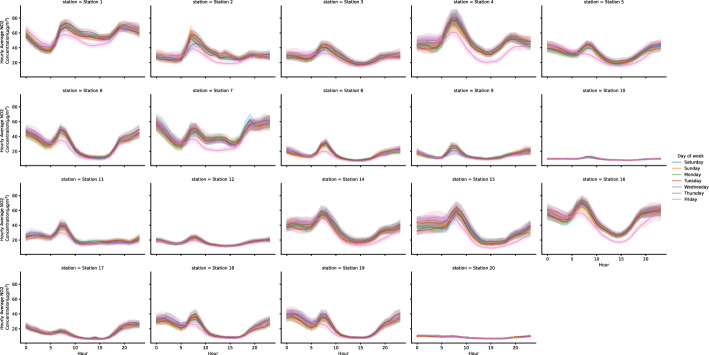
NO$_{2}$ temporal variation Temporal hourly and daily of the week NO$_{2}$ concentration from variations for the 19 stations from 2019 to 2020. The hourly concentration of NO$_{2}$ is highest in the early morning and lowest in the mid-afternoon. The station geo-location have an impact on the pollutant concentrates, the highest concentration found in the traffic area and lowers in the rural area

The highest mean NO$_{2}$ concentration was reported in 2019 from station 1 (56.44 $\mu$g/m$^{3}$; urban traffic), while the lowest average values were reported in station 20 (8.60 $\mu$g/m$^{3}$, rural background) for the same year. From Fig. 3, we observed that NO$_{2}$ concentration is lower in stations 10 and 20 (rural background) and higher in stations 1,4, 15, and 16 (all of them are in the Abu Dhabi Capital Region). There is an apparent annual periodicity in the NO$_{2}$ emission; a high NO$_{2}$ emission is found early in the year, reduced during the summertime, and increased again after the summertime. The nonparametric seasonal Mann-Kendall trend test and Sen’s slope estimator (Table 2) reported a significant decrease in the annual trend (p<0.05) of the NO$_{2}$ concentration for most of the stations, however, a significant increase is reported for stations 5 and 9. Figure 4 presents the temporal hourly and daily NO$_{2}$ concentration variations of the 19 stations during 2019 and 2020. Overall, NO$_{2}$ concentrations exhibit a similar pattern across the different stations. During 2019 and 2022, Friday and Saturday were for the weekend, while Sunday until Thursday were the working days. The hourly emission of NO$_{2}$ is highest in the early morning from 5:00 am to 10:00 am and lowest in the mid-afternoon from 2:00 pm to 4:00 pm. For the day of the week temporal variation, we can notice NO$_{2}$ production is lower during weekends, especially Friday, the first day of the weekend, and increases during the working days. For stations 10 and 20, the temporal hourly and daily NO$_{2}$ concentration is flattening since those regions represent a Rural Background consisting mainly of a desert; therefore, not so many human activities that contribute to increase the concentration of the pollutants.

### Predictive deep learning models

**Fig. 5:**
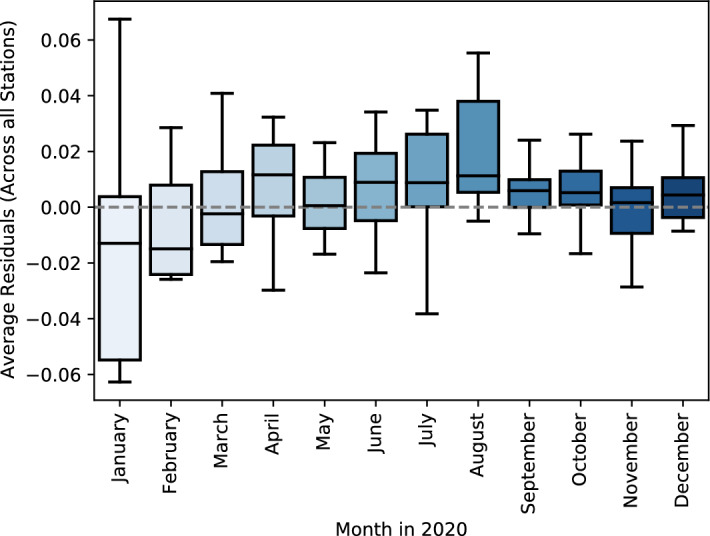
TSTransformer Residuals: TSTransformer Model average residuals of NO$_{2}$ concentrations for each month in 2020 (Across all the stations) on the testing set

Using time series cross-validation, we trained the models using a series of training sets for each model to forecast NO$_{2}$ concentration for one month ahead (the observation that forms the test set). The model performance metrics were computed by averaging the model performance over the test sets. We trained several state-of-the-art deep learning models for sequence data, namely, MiniRocket, ResNet for time series, XceptionTime, InceptionTime and Transformer for time series. In this study, we trained the models using data from different monitoring stations which exhibit various environmental assessment points. Table 3 presents the performance of the trained models in the testing set. Overall, the Transformer-based deep learning model reports the best performance in the unseen data compared with other deep learning models: MiniRocket, ResNet for time series, XceptionTime, and InceptionTime. For the Transformer model, the minimum RMSE is 0.00102 (±0.00071) reported by station 12 with MAE: 0.02488 (±0.0091) and MSE: 0.03018 (±0.01055). The same model reports the maximum RMSE (0.01468 (±0.03387)) for station 10 with MAE: 0.06707 (± 0.06861) and MSE: 0.08505 (±0.08629). The performance of the Transformer is outperform other models in all the stations. It is important to emphasize that R$^{2}$ is a measure of goodness-of-fit, not a measure of model’s predictive capability [34]; the high R$^{2}$ value for the model explained by the increase in the variance of the time series; in which having a larger variance in the time series can cause the R$^{2}$ value to be closed to one, and can be deceiving when calculating the model quality. Finally for model interpenetration, we used permutation feature importance for interpreting the transformer model; Table 4 presents that the model assigned a high weights to the fourth day for predict the future NO$_{2}$ value. In Fig. 5, we visualize the Transformer model’s average residual performance by calculating the difference between the predicted and actual values. Transformer-based models show good performance during fall and bad during summertime. During the Covid-19 period, the model performed severely due to the sudden change in the trends.

**Table 3:** The performance of different deep learning models for NO$_{2}$ emission prediction in the testing set

	MAE						MSE					
Station	M0	M1	M2	M3	M4	M5	M0	M1	M2	M3	M4	M5
Station 1	0.19 (± 0.18)	0.26 (± 0.19)	0.21 (± 0.07)	0.24 (± 0.16)	0.04 (± 0.04)	0.7 (± 0.55)	0.22 (± 0.18)	0.31 (± 0.21)	0.26 (± 0.08)	0.28 (± 0.16)	0.06 (± 0.04)	0.79 (± 0.54)
Station 2	0.17 (± 0.18)	0.27 (± 0.24)	0.24 (± 0.16)	0.24 (± 0.17)	0.05 (± 0.04)	0.69 (± 0.21)	0.21 (± 0.21)	0.32 (± 0.3)	0.28 (± 0.16)	0.29 (± 0.22)	0.07 (± 0.06)	0.81 (± 0.2)
Station 3	0.13 (± 0.15)	0.3 (± 0.28)	0.18 (± 0.15)	0.17 (± 0.15)	0.04 (± 0.04)	0.49 (± 0.28)	0.16 (± 0.17)	0.37 (± 0.35)	0.21 (± 0.17)	0.22 (± 0.18)	0.05 (± 0.05)	0.59 (± 0.27)
Station 4	0.1 (± 0.07)	0.25 (± 0.16)	0.17 (± 0.09)	0.16 (± 0.1)	0.05 (± 0.05)	0.31 (± 0.26)	0.12 (± 0.09)	0.29 (± 0.17)	0.21 (± 0.1)	0.18 (± 0.12)	0.06 (± 0.08)	0.38 (± 0.27)
Station 5	0.09 (± 0.08)	0.22 (± 0.14)	0.21 (± 0.14)	0.13 (± 0.09)	0.05 (± 0.05)	0.28 (± 0.14)	0.11 (± 0.11)	0.27 (± 0.18)	0.26 (± 0.16)	0.16 (± 0.13)	0.06 (± 0.06)	0.35 (± 0.15)
Station 6	0.11 (± 0.08)	0.19 (± 0.22)	0.16 (± 0.04)	0.17 (± 0.12)	0.04 (± 0.03)	0.54 (± 0.24)	0.14 (± 0.09)	0.24 (± 0.25)	0.2 (± 0.05)	0.21 (± 0.13)	0.05 (± 0.04)	0.66 (± 0.26)
Station 7	0.08 (± 0.05)	0.23 (± 0.08)	0.12 (± 0.04)	0.11 (± 0.07)	0.03 (± 0.02)	0.48 (± 0.18)	0.1 (± 0.07)	0.28 (± 0.09)	0.14 (± 0.05)	0.15 (± 0.09)	0.04 (± 0.03)	0.55 (± 0.17)
Station 8	0.08 (± 0.1)	0.14 (± 0.13)	0.16 (± 0.06)	0.11 (± 0.07)	0.04 (± 0.04)	0.37 (± 0.17)	0.1 (± 0.12)	0.16 (± 0.15)	0.2 (± 0.06)	0.14 (± 0.1)	0.04 (± 0.05)	0.48 (± 0.18)
Station 9	0.13 (± 0.1)	0.24 (± 0.17)	0.19 (± 0.12)	0.21 (± 0.19)	0.04 (± 0.04)	0.32 (± 0.18)	0.16 (± 0.14)	0.3 (± 0.21)	0.25 (± 0.16)	0.25 (± 0.22)	0.05 (± 0.06)	0.44 (± 0.22)
Station 10	0.18 (± 0.16)	0.26 (± 0.17)	0.19 (± 0.07)	0.21 (± 0.12)	0.07 (± 0.07)	0.63 (± 0.43)	0.23 (± 0.22)	0.31 (± 0.21)	0.23 (± 0.09)	0.27 (± 0.16)	0.09 (± 0.09)	0.76 (± 0.42)
Station 11	0.12 (± 0.08)	0.18 (± 0.16)	0.21 (± 0.05)	0.21 (± 0.11)	0.04 (± 0.02)	0.36 (± 0.15)	0.15 (± 0.1)	0.23 (± 0.2)	0.25 (± 0.06)	0.26 (± 0.13)	0.04 (± 0.03)	0.51 (± 0.16)
Station 12	0.07 (± 0.03)	0.17 (± 0.1)	0.13 (± 0.03)	0.08 (± 0.04)	0.02 (± 0.01)	0.56 (± 0.25)	0.09 (± 0.05)	0.2 (± 0.11)	0.15 (± 0.04)	0.1 (± 0.05)	0.03 (± 0.01)	0.67 (± 0.27)
Station 14	0.09 (± 0.06)	0.24 (± 0.21)	0.18 (± 0.11)	0.14 (± 0.11)	0.03 (± 0.02)	0.49 (± 0.27)	0.1 (± 0.07)	0.29 (± 0.25)	0.21 (± 0.13)	0.17 (± 0.13)	0.03 (± 0.02)	0.59 (± 0.26)
Station 15	0.07 (± 0.06)	0.2 (± 0.15)	0.14 (± 0.05)	0.11 (± 0.08)	0.02 (± 0.01)	0.61 (± 0.22)	0.09 (± 0.07)	0.24 (± 0.18)	0.17 (± 0.06)	0.14 (± 0.11)	0.03 (± 0.01)	0.7 (± 0.21)
Station 16	0.11 (± 0.06)	0.24 (± 0.15)	0.18 (± 0.07)	0.16 (± 0.09)	0.04 (± 0.01)	0.39 (± 0.27)	0.14 (± 0.08)	0.28 (± 0.17)	0.22 (± 0.08)	0.2 (± 0.11)	0.05 (± 0.02)	0.5 (± 0.28)
Station 17	0.08 (± 0.04)	0.15 (± 0.15)	0.17 (± 0.06)	0.16 (± 0.09)	0.03 (± 0.01)	0.48 (± 0.29)	0.1 (± 0.06)	0.19 (± 0.19)	0.21 (± 0.08)	0.19 (± 0.11)	0.04 (± 0.01)	0.63 (± 0.29)
Station 18	0.11 (± 0.11)	0.17 (± 0.24)	0.18 (± 0.09)	0.17 (± 0.17)	0.04 (± 0.04)	0.45 (± 0.24)	0.14 (± 0.14)	0.21 (± 0.28)	0.21 (± 0.11)	0.21 (± 0.2)	0.05 (± 0.05)	0.56 (± 0.23)
Station 19	0.09 (± 0.08)	0.22 (± 0.13)	0.14 (± 0.03)	0.15 (± 0.09)	0.03 (± 0.01)	0.47 (± 0.26)	0.11 (± 0.09)	0.26 (± 0.16)	0.18 (± 0.04)	0.18 (± 0.1)	0.04 (± 0.02)	0.55 (± 0.26)
Station 20	0.11 (± 0.05)	0.28 (± 0.12)	0.16 (± 0.05)	0.15 (± 0.08)	0.03 (± 0.01)	0.39 (± 0.26)	0.13 (± 0.06)	0.34 (± 0.15)	0.19 (± 0.06)	0.19 (± 0.1)	0.04 (± 0.01)	0.47 (± 0.28)
Average	0.11 (±0.09)	0.22 (±0.17)	0.17 (±0.08)	0.16 (±0.11)	0.04 (±0.03)	0.47 (±0.26)	0.14 (±0.11)	0.27 (±0.20)	0.21 (±0.09)	0.20 (±0.13 )	0.05 (±0.04)	0.58 (±0.26)

	R2						RMSE					
Station 1	M0	M1	M2	M3	M4	M5	M0	M1	M2	M3	M4	M5
Station 2	0.38 (± 1.35)	0.56 (± 0.33)	0.65 (± 0.28)	0.35 (± 1.19)	0.98 (± 0.05)	−4.83 (± 9.38)	0.08 (± 0.13)	0.14 (± 0.17)	0.07 (± 0.04)	0.11 (± 0.12)	0.0 (± 0.01)	0.91 (± 1.06)
Station 3	0.9 (± 0.09)	0.71 (± 0.31)	0.79 (± 0.15)	0.82 (± 0.12)	0.99 (± 0.01)	−1.43 (± 1.82)	0.09 (± 0.18)	0.19 (± 0.25)	0.1 (± 0.12)	0.13 (± 0.19)	0.01 (± 0.01)	0.69 (± 0.32)
Station 4	0.92 (± 0.09)	0.54 (± 0.4)	0.84 (± 0.12)	0.85 (± 0.12)	0.99 (± 0.01)	−1.71 (± 3.32)	0.06 (± 0.1)	0.25 (± 0.4)	0.07 (± 0.11)	0.08 (± 0.12)	0.0 (± 0.01)	0.43 (± 0.32)
Station 5	0.93 (± 0.07)	0.55 (± 0.34)	0.79 (± 0.15)	0.85 (± 0.11)	0.98 (± 0.04)	−0.92 (± 4.43)	0.02 (± 0.03)	0.12 (± 0.13)	0.05 (± 0.06)	0.05 (± 0.06)	0.01 (± 0.03)	0.22 (± 0.34)
Station 6	0.92 (± 0.09)	0.52 (± 0.36)	0.51 (± 0.54)	0.87 (± 0.11)	0.97 (± 0.05)	−0.2 (± 1.39)	0.02 (± 0.05)	0.11 (± 0.17)	0.09 (± 0.11)	0.04 (± 0.07)	0.01 (± 0.01)	0.15 (± 0.1)
Station 7	0.95 (± 0.04)	0.85 (± 0.2)	0.9 (± 0.05)	0.9 (± 0.08)	1.0 (± 0.01)	−0.32 (± 1.04)	0.03 (± 0.04)	0.12 (± 0.26)	0.04 (± 0.02)	0.06 (± 0.08)	0.0 (± 0.01)	0.51 (± 0.32)
Station 8	0.9 (± 0.1)	0.22 (± 0.36)	0.79 (± 0.13)	0.79 (± 0.17)	0.98 (± 0.02)	−2.83 (± 3.58)	0.01 (± 0.02)	0.09 (± 0.05)	0.02 (± 0.02)	0.03 (± 0.03)	0.0 (± 0.0)	0.33 (± 0.19)
Station 9	0.96 (± 0.06)	0.85 (± 0.21)	0.82 (± 0.13)	0.92 (± 0.06)	0.99 (± 0.01)	−0.31 (± 1.43)	0.03 (± 0.06)	0.05 (± 0.08)	0.04 (± 0.03)	0.03 (± 0.05)	0.0 (± 0.01)	0.26 (± 0.17)
Station 10	0.95 (± 0.05)	0.72 (± 0.28)	0.84 (± 0.16)	0.86 (± 0.13)	0.99 (± 0.01)	0.34 (± 0.62)	0.05 (± 0.08)	0.14 (± 0.15)	0.09 (± 0.13)	0.11 (± 0.18)	0.01 (± 0.02)	0.24 (± 0.28)
Station 11	0.86 (± 0.17)	0.78 (± 0.15)	0.82 (± 0.19)	0.75 (± 0.37)	0.98 (± 0.03)	−2.59 (± 5.73)	0.1 (± 0.23)	0.14 (± 0.18)	0.06 (± 0.05)	0.1 (± 0.11)	0.01 (± 0.03)	0.74 (± 0.8)
Station 12	0.96 (± 0.05)	0.9 (± 0.13)	0.9 (± 0.06)	0.9 (± 0.07)	1.0 (± 0.0)	0.61 (± 0.23)	0.03 (± 0.04)	0.09 (± 0.12)	0.07 (± 0.03)	0.09 (± 0.07)	0.0 (± 0.0)	0.28 (± 0.18)
Station 13	0.96 (± 0.04)	0.79 (± 0.17)	0.88 (± 0.06)	0.95 (± 0.02)	0.99 (± 0.01)	−1.83 (± 2.23)	0.01 (± 0.01)	0.05 (± 0.05)	0.02 (± 0.01)	0.01 (± 0.01)	0.0 (± 0.0)	0.52 (± 0.3)
Station 14	0.94 (± 0.05)	0.62 (± 0.33)	0.78 (± 0.16)	0.86 (± 0.13)	0.99 (± 0.01)	−5.16 (± 13.18)	0.02 (± 0.02)	0.14 (± 0.22)	0.06 (± 0.08)	0.05 (± 0.07)	0.0 (± 0.0)	0.41 (± 0.3)
Station 15	0.95 (± 0.03)	0.64 (± 0.28)	0.79 (± 0.13)	0.86 (± 0.11)	0.99 (± 0.0)	−3.96 (± 4.17)	0.01 (± 0.02)	0.09 (± 0.11)	0.03 (± 0.02)	0.03 (± 0.04)	0.0 (± 0.0)	0.54 (± 0.29)
Station 16	0.93 (± 0.04)	0.66 (± 0.25)	0.81 (± 0.08)	0.86 (± 0.08)	0.99 (± 0.0)	−0.25 (± 1.33)	0.02 (± 0.03)	0.11 (± 0.11)	0.06 (± 0.05)	0.05 (± 0.05)	0.0 (± 0.0)	0.33 (± 0.3)
Station 17	0.97 (± 0.02)	0.87 (± 0.16)	0.86 (± 0.07)	0.9 (± 0.06)	0.99 (± 0.01)	−1.06 (± 2.93)	0.01 (± 0.02)	0.07 (± 0.1)	0.05 (± 0.04)	0.05 (± 0.05)	0.0 (± 0.0)	0.47 (± 0.38)
Station 18	0.95 (± 0.05)	0.86 (± 0.21)	0.88 (± 0.05)	0.89 (± 0.11)	0.99 (± 0.01)	−0.46 (± 1.8)	0.04 (± 0.09)	0.12 (± 0.29)	0.06 (± 0.08)	0.08 (± 0.17)	0.01 (± 0.01)	0.36 (± 0.28)
Station 19	0.96 (± 0.04)	0.68 (± 0.31)	0.87 (± 0.08)	0.88 (± 0.07)	0.99 (± 0.0)	−0.7 (± 1.91)	0.02 (± 0.04)	0.1 (± 0.1)	0.03 (± 0.01)	0.04 (± 0.05)	0.0 (± 0.0)	0.37 (± 0.29)
Station 20	0.92 (± 0.07)	0.46 (± 0.24)	0.83 (± 0.06)	0.84 (± 0.1)	0.99 (± 0.01)	−0.47 (± 1.29)	0.02 (± 0.02)	0.14 (± 0.11)	0.04 (± 0.02)	0.05 (± 0.04)	0.0 (± 0.0)	0.3 (± 0.33)
Average	0.91 (±0.13)	0.67 (±0.26)	0.81 (±0.14)	0.84 (±0.17)	0.99 (±0.02)	−1.48 (±3.25 )	0.04 (±0.06)	0.12 (±0.16)	0.06 (±0.05)	0.06 (±0.08)	0.00 (±0.01)	0.42 (±0.34)

M0: InceptionTime; M1: LSTM; M2: MiniRocket, M3: ResNet, M4: TSTransformer and M5: XceptionTime

**Table 4:** Feature Importance of the Transformer model using Cross-validation: The permutation feature importance reported the decrease in the MAE score when the single value is randomly shuffled. The most important features are the one with a higher values

Station	Lag 10	Lag 9	Lag 8	Lag 7	Lag 6	Lag 5	Lag 4	Lag 3	Lag 2	Lag 1
Station 1	0.02 (± 0.01)	0.02 (± 0.01)	0.02 (± 0.01)	0.02 (± 0.01)	0.02 (± 0.01)	0.02 (± 0.01)	1.06 (± 0.03)	0.02 (± 0.01)	0.02 (± 0.01)	0.02 (± 0.01)
Station 2	0.01 (± 0.0)	0.01 (± 0.0)	0.01 (± 0.0)	0.01 (± 0.0)	0.01 (± 0.0)	0.01 (± 0.0)	0.92 (± 0.04)	0.01 (± 0.0)	0.01 (± 0.0)	0.01 (± 0.0)
Station 3	0.01 (± 0.0)	0.01 (± 0.0)	0.01 (± 0.0)	0.01 (± 0.0)	0.01 (± 0.0)	0.01 (± 0.0)	1.03 (± 0.03)	0.01 (± 0.0)	0.01 (± 0.01)	0.01 (± 0.0)
Station 4	0.01 (± 0.0)	0.01 (± 0.0)	0.01 (± 0.0)	0.01 (± 0.0)	0.01 (± 0.0)	0.01 (± 0.01)	1.0 (± 0.04)	0.01 (± 0.01)	0.01 (± 0.01)	0.01 (± 0.0)
Station 5	0.02 (± 0.01)	0.02 (± 0.0)	0.02 (± 0.01)	0.02 (± 0.01)	0.03 (± 0.01)	0.02 (± 0.01)	0.89 (± 0.05)	0.02 (± 0.01)	0.02 (± 0.01)	0.02 (± 0.01)
Station 6	0.01 (± 0.0)	0.01 (± 0.0)	0.01 (± 0.0)	0.01 (± 0.0)	0.01 (± 0.0)	0.01 (± 0.0)	1.06 (± 0.03)	0.01 (± 0.0)	0.01 (± 0.0)	0.01 (± 0.0)
Station 7	0.03 (± 0.01)	0.03 (± 0.01)	0.03 (± 0.01)	0.03 (± 0.01)	0.03 (± 0.01)	0.04 (± 0.01)	0.94 (± 0.03)	0.04 (± 0.02)	0.04 (± 0.01)	0.03 (± 0.01)
Station 8	0.01 (± 0.0)	0.01 (± 0.0)	0.01 (± 0.0)	0.01 (± 0.0)	0.01 (± 0.0)	0.01 (± 0.0)	1.02 (± 0.03)	0.01 (± 0.0)	0.01 (± 0.0)	0.01 (± 0.0)
Station 9	0.01 (± 0.0)	0.01 (± 0.0)	0.01 (± 0.0)	0.01 (± 0.0)	0.01 (± 0.0)	0.01 (± 0.0)	1.05 (± 0.05)	0.01 (± 0.01)	0.01 (± 0.0)	0.01 (± 0.0)
Station 10	0.01 (± 0.01)	0.01 (± 0.01)	0.01 (± 0.0)	0.01 (± 0.01)	0.01 (± 0.01)	0.02 (± 0.01)	1.02 (± 0.05)	0.01 (± 0.01)	0.01 (± 0.0)	0.01 (± 0.0)
Station 11	0.01 (± 0.0)	0.01 (± 0.0)	0.01 (± 0.0)	0.01 (± 0.0)	0.01 (± 0.0)	0.01 (± 0.0)	1.05 (± 0.03)	0.01 (± 0.0)	0.01 (± 0.0)	0.01 (± 0.0)
Station 12	0.02 (± 0.01)	0.01 (± 0.0)	0.01 (± 0.0)	0.02 (± 0.0)	0.02 (± 0.01)	0.02 (± 0.01)	1.0 (± 0.01)	0.02 (± 0.01)	0.02 (± 0.01)	0.02 (± 0.01)
Station 14	0.01 (± 0.0)	0.02 (± 0.0)	0.01 (± 0.0)	0.01 (± 0.0)	0.02 (± 0.0)	0.02 (± 0.0)	0.93 (± 0.04)	0.01 (± 0.0)	0.01 (± 0.0)	0.01 (± 0.0)
Station 15	0.02 (± 0.01)	0.02 (± 0.0)	0.01 (± 0.0)	0.01 (± 0.0)	0.02 (± 0.01)	0.01 (± 0.0)	1.03 (± 0.03)	0.02 (± 0.01)	0.01 (± 0.0)	0.01 (± 0.0)
Station 16	0.01 (± 0.0)	0.01 (± 0.0)	0.02 (± 0.01)	0.02 (± 0.01)	0.01 (± 0.01)	0.02 (± 0.01)	1.07 (± 0.02)	0.02 (± 0.01)	0.02 (± 0.0)	0.01 (± 0.0)
Station 17	0.01 (± 0.0)	0.01 (± 0.0)	0.01 (± 0.0)	0.01 (± 0.0)	0.01 (± 0.0)	0.01 (± 0.0)	1.04 (± 0.04)	0.01 (± 0.0)	0.01 (± 0.0)	0.01 (± 0.0)
Station 18	0.01 (± 0.0)	0.01 (± 0.0)	0.01 (± 0.0)	0.01 (± 0.0)	0.01 (± 0.0)	0.01 (± 0.01)	1.03 (± 0.04)	0.01 (± 0.0)	0.01 (± 0.0)	0.01 (± 0.0)
Station 19	0.01 (± 0.01)	0.01 (± 0.0)	0.01 (± 0.0)	0.01 (± 0.0)	0.01 (± 0.0)	0.01 (± 0.0)	1.0 (± 0.04)	0.01 (± 0.0)	0.01 (± 0.0)	0.01 (± 0.01)
Station 20	0.02 (± 0.01)	0.03 (± 0.0)	0.03 (± 0.01)	0.02 (± 0.01)	0.03 (± 0.01)	0.03 (± 0.01)	1.07 (± 0.04)	0.03 (± 0.01)	0.03 (± 0.01)	0.03 (± 0.01)

## Discussion

The atmospheric model and its composition research are receiving increased interest among the scientific community to tackle major global challenges such as climate change, air quality, urbanization, etc. [2]. One of the important atmospheric research tracks is a short-term and annual average air quality forecast that supports the decision makers to adopt the appropriate regulations and laws for improving air quality and public health [2, 8]. Air quality prediction is considered a challenging task since air pollutants concentration is governed by environmental and physical factors such as meteorological factors, traffic pollution, and industrial emissions that vary across time and space [8, 16].

This study aims to investigate the NO$_{2}$ patterns in the UAE and implement several state-of-the-art deep learning models, MiniRocket, ResNet for time series, XceptionTime, InceptionTime, and Transformer, for future NO$_{2}$ forecasting using historical data. The data was collected from different monitoring stations that distributed and exhibited various environmental assessment regions across Abu-Dhabi: urban traffic, urban background, rural traffic, rural background, rural industrial, suburban background, and suburban industrial. The UAE’s primary sources of NO$_{2}$ emissions come from the production and refining of oil and gas, power generation, and water desalination, while the second source is from vehicles and ships [35]. From Fig. 3, Station 1, which has the highest pollutant emissions located in an urban area with traffic; so, the vehicle emission explains this increase in the pollutant, while station 20, which has the lowest NO$_{2}$ emission, is a rural area. We can conclude that traffic is one of the primary sources of NO$_{2}$ concentrations. In general, we notice a reduction in the NO$_{2}$ pollutants during summertime; the latter is explained by the involvement of NO$_{2}$ in producing the ground-level ozone (O$_{3}$) pollutant during summer. From a chemical point of view, NO$_{2}$ and carbon monoxide are photochemical reactions combined with solar radiation to produce O$_{3}$; most of these reactions happen during summer [8, 36]. Most stations exhibit a decrease in the annual NO$_{2}$ level except for stations 5 and 9. The reduction in the pollutant trend of most stations reflects the UAE’s efforts to improve the air quality. Some of the notable efforts are: launching the National Air Quality Platform [37] for the researchers to study the different factors that affect pollutant levels in the region; collaborating with several federal and local government agencies to create joint initiatives and best practices to improve air quality; encouraging the society to reduce the pollutants emission and adopt environment-friendly practices [38]. In 2020 and with the spread of coronavirus disease (COVID-19), UAE took several measures to control the spread of the disease, such as lockdown and social distancing; those measures significantly reduced the NO$_{2}$ emission, as reported in [15]. This study confirmed the same findings (Fig. 3); NO$_{2}$ concentration was decreased from the End of March 2020 until early July 2020; due to mobility restrictions (traffic and vehicles usage reduction); these findings are valid for all the stations except for the Al Dhafra Region stations (a vast expanse of the desert). For the day of the week temporal pattern, the pollutant emission increases in the early morning, especially during the working days, due to motor vehicle movement and traffic. The daily pattern is expected to change; as in 2022, all the federal government entities in the UAE operates from Monday until half day on Friday, with the weekend starting from the second half of Friday until Sunday [39].

For the predictive model, the Transformer-based deep learning model outperforms other models to forecast daily NO$_{2}$ concentration for one month ahead; the best performance was reported in station 12 (MAE:0.02488 (±0.0091), MSE:0.03018 (±0.01055), RMSE: 0.00102 (±0.00071), R$^{2}$: 0.99376 (±0.00692)). While XceptionTime reported the worst results across all the stations. The transformer model’s superiority is explained by the attention head, which is a powerful technique to effectively learn the new representation of the sequence data by relating different positions in the sequence. The overall performance of the Transformer model indicates it is capable of capturing the pollutants’ daily and weekly cycle patterns exhibited in the pollutants trend (Figs. 3, 4). One of the limitations is the existence of the none meteorological interventions such as Covid-19, which lower the model performance. Overall, the model’s performance is good when trained using different time series, which exhibited different variability of NO$_{2}$ concentration. In cooperated with other pollutants data could be improved the overall model performance.

Previous studies applied different ML and AI models to improve the NO$_{2}$ concentration prediction. One of the earliest studies [13] used cluster-based bagging machine learning models to predict NO$_{2}$ concentration for the state of California. The model was trained using historical NO$_{2}$ data, traffic-related NO$_{x}$ modeled by CALINE4 dispersion model, traffic density, distance to shoreline and roadways, air temperature, population density, humidity, precipitation, and wind speed. The model reported (R$^{2}$=0.87$-$0.9, RMSE=0.21$-$0.27). Another study utilized Tehran metropolis air quality data, Iran [1], to build a multi-linear regression (MLR) and multilayer perceptron model (MLP) models and used the trained model to forecast future NO$_{2}$ concentration. The study improved NO$_{2}$ prediction by incorporating additional features to the model, such as traffic and green space information, the day of the week, and meteorological parameters. The MLP model reported (R$^{2}$ = 0.89, RMSE= 0.32) which outperform MLR (R$^{2}$ = 0.81, RMSE= 13.151). A third study used data from 35 monitoring stations in Beijing, China [8], to propose a novel multi-output and multi-index supervised learning model based on LSTM. The model predicts several air pollutants: PM$_{2.5}$, CO, NO$_{2}$, O$_{3}$, and SO$_{2}$, using meteorological and gaseous pollutant data from the closest five neighbors' stations-as input to the model. The model best performance reported for NO$_{2}$ prediction was (R$^{2}$ = 0.875, RMSE= 9.688, MAE = 6.47). Another study [9]also used China monitoring stations data; it proposed a novel method that integrates discrete wavelet transformation for time series decomposition followed by training LSTM Network to improve NO$_{2}$ level prediction. The model inputs multiple covariates: PM$_{2.5}$, PM$_{10}$, NO$_{2}$, SO$_{2}$, O$_{3}$, CO, wind speed, temperature, and weather conditions. The reported performance of the proposed model in the unseen data is MAE =4.3377 and RMSE = 5.9291. Finally, a recent study [3], using Madrid, Spain, data proposed several deep learning models, namely LSTM and ConvLSTM and Bidirectional convolutional LSTM (BiConvLSTM), to predict NO$_{2}$ level. The model inputs NO$_{2}$ historical information, ultraviolet radiation, wind speed, wind direction, temperature, relative humidity, barometric pressure, solar irradiance, precipitation and traffic intensity, occupancy time, and average traffic speed of 24 monitoring stations. It found that BiConvLSTM (RMSE = 19.14, MAE =13.06) outperform LSTM (RMSE =38.89, MAE =32.17) and ConvLSTM (RMSE =32.95, MAE =32.04) for NO$_{2}$ prediction.

Even though there are several efforts to improve the accuracy of future NO$_{2}$ level prediction using machine learning and deep learning models, the best reported R$^{2}$ and RMSE from the previous published works range from 0.87 to 0.9 and from 0.21 to 19.14, respectively. NO$_{2}$ prediction is a complex task, that is why all the previous works integrate environmental and physical factors such as traffic data, wind speed, wind direction, humidity, air temperature, and air pressure to reach the best-reported results.

By validating that NO$_{2}$ exhibits a periodic pattern, as reported in Fig. 3 and Fig. 4, we implemented several state-of-the-art deep learning models for sequence data using NO$_{2}$ historical information only to predict future NO$_{2}$ levels. This study proves that Transformer deep learning models are superior to learning the temporal data representation to make precision forecasting compared to statistical models, machine learning, and early neural network models. Even though there is a change in the NO$_{2}$ pattern due to the COVID-19 pandemic, the models reported a reasonable performance in comparison with what had been reported in the literature so far.

## Conclusion

In this study, we implement various state-of-the-art deep learning models to predict the NO$_{2}$ emissions using pollutant univariate historical data; the models were tested across different monitoring stations in Abu-Dhabi that exhibit various environmental assessment points. We reveal a general decrease in the NO$_{2}$ annual patterns for most stations, and we confirm the impact of the COVID-19 lockdown on reducing the NO$_{2}$. Using the Transformer deep learning model for time series data, we improved the accuracy of NO$_{2}$ forecasting. Our findings outperformed all the results reported in the literature for the same task using only NO$_{2}$ historical data. This study trained and validated the models on a particular type of air pollutant (NO$_{2}$); however, several hazardous pollutants are of significant importance for atmospheric management decisions, such as PM$_{2.5}$, O$_{3}$, etc. Future work will be directed toward implementing and testing the different deep learning models to predict different air pollutants concentrations; predicting NO$_{2}$ concentrations at hourly intervals and using the deep learning techniques to reveal the association between different pollutants such as NO$_{2}$ and ozone production. Moreover, this study implemented different models for each station (in total we trained 1,368: 6 models, 19 stations, 12 months prediction for cross-validation), which are computationally time-consuming and expensive. Investigating the capabilities of training a single model and adopt it(transfer learning) to all other stations will be considered to reduce the computation resource.

## Supplementary Information

**Additional file 1**: **Table S1**: Missing Value Percentages in the 20 UAE monitoring stations. **Figure S1**. Station 1 NO2 concentration: A)Missing values distribution: The missing regions are highlighted. B) Missing values imputation: visualization of missing value replacements. **Figure S2**: Station 2 NO2 concentration: A)Missing values distribution: The missing regions are highlighted. B) Missing values imputation: visualization of missing value replacements. **Figure S3**: Station 3 NO2 concentration: A)Missing values distribution: The missing regions are highlighted. B) Missing values imputation: visualization of missing value replacements.**Figure S4**: Station 4 NO2 concentration: A)Missing values distribution: The missing regions are highlighted. B) Missing values imputation: visualization of missing value replacements. **Figure S5**: Station 5 NO2 concentration: A)Missing values distribution: The missing regions are highlighted. B) Missing values imputation: visualization of missing value replacements. **Figure S6**: Station 6 NO2 concentration: A)Missing values distribution: The missing regions are highlighted. B) Missing values imputation: visualization of missing value replacements. **Figure S7**: Station 7 NO2 concentration: A)Missing values distribution: The missing regions are highlighted. B) Missing values imputation: visualization of missing value replacements. **Figure S8**: Station 8 NO2 concentration: A)Missing values distribution: The missing regions are highlighted. B) Missing values imputation: visualization of missing value replacements. **Figure S9**: Station 9 NO2 concentration: A)Missing values distribution: The missing regions are highlighted. B) Missing values imputation: visualization of missing value replacements. **Figure S10**: Station 10 NO2 concentration: A)Missing values distribution: The missing regions are highlighted. B) Missing values imputation: visualization of missing value replacements. **Figure S11**: Station 11 NO2 concentration: A)Missing values distribution: The missing regions are highlighted. B) Missing values imputation: visualization of missing value replacements.**Figure S12**: Station 12 NO2 concentration: A)Missing values distribution: The missing regions are highlighted. B) Missing values imputation: visualization of missing value replacements. **Figure S13**: Station 14 NO2 concentration: A)Missing values distribution: The missing regions are highlighted. B) Missing values imputation: visualization of missing value replacements. **Figure S14**: Station 15 NO2 concentration: A)Missing values distribution: The missing regions are highlighted. B) Missing values imputation: visualization of missing value replacements. **Figure S15**: Station 16 NO2 concentration: A)Missing values distribution: The missing regions are highlighted. B) Missing values imputation: visualization of missing value replacements. **Figure S16**: Station 17 NO2 concentration: A)Missing values distribution: The missing regions are highlighted. B) Missing values imputation: visualization of missing value replacements. **Figure S17**: Station 18 NO2 concentration: A)Missing values distribution: The missing regions are highlighted. B) Missing values imputation: visualization of missing value replacements. **Figure S18**: Station 19 NO2 concentration: A)Missing values distribution: The missing regions are highlighted. B) Missing values imputation: visualization of missing value replacements. **Figure S19**: Station 20 NO2 concentration: A)Missing values distribution: The missing regions are highlighted. B) Missing values imputation: visualization of missing value replacements

## Data Availability

The data that support the findings of this study are available from Environment Agency - Abu Dhabi Air Quality Data but restrictions apply to the availability of these data, which were used under license for the current study, and so are not publicly available. Data are however available by submit a request to Environment Agency - Abu Dhabi Air Quality Data.

## Abbreviations

AL: Artificial intelligence
AQG: Air Quality Guidelines
AR: Auto-Regressive model
ARIMA: Auto-Regressive Integrated Moving Average model
BiConvLSTM: Bidirectional Convolutional LSTM
CO: Carbon Monoxide
EAD: Environment Agency-Abu Dhabi
EWMA: Exponential Weighted Moving Average
H$$_{2}$$S: Hydrogen Sulfide
LSTM: Long Short-Term Memory
MA: Moving Average model
MAE: Mean Absolute Error
MiniRocket: MINImally RandOm Convolutional KErnel Transform
ML: Machine Learning
MLP: MultiLayer Perceptron model
MLR: Multilinear Regression
MSE: Mean Square Error
NO$_{2}$: Nitrogen Dioxide
O$$_{3}$$: Ozone
PM: Particulate Matter
R$^{2}$: Coefficient of determination
ReLU: Rectified Linear Unit
ResNet: Residual Network
RMSE: Root Means Square Error
RNN: Recurrent Neural Network
SARIMA: Seasonal ARIMA
SO$_{2}$: Sulfur Dioxide
UAE: United Arab Emirates
WHO: World Health Organization
