# Supplementary material for: Artificial intelligence for improving Nitrogen Dioxide forecasting of Abu Dhabi environment agency ground-based stations
Source: J Big Data. 2023 Jun 2;10(1):92. doi: 10.1186/s40537-023-00754-z (PMC10236404; doi:10.1186/s40537-023-00754-z)
Supplement: Supplementary file 1 — Additional file 1: Table S1: Missing Value Percentages in the 20 UAE monitoring stations. Figure S1. Station 1 NO2 concentration: A)Missing values distribution: The missing regions are highlighted. B) Missing values imputation: visualization of missing value replacements. Figure S2: Station 2 NO2 concentration: A)Missing values distribution: The missing regions are highlighted. B) Missing values imputation: visualization of missing value replacements. Figure S3: Station 3 NO2 concentration: A)Missing values distribution: The missing regions are highlighted. B) Missing values imputation: visualization of missing value replacements.Figure S4: Station 4 NO2 concentration: A)Missing values distribution: The missing regions are highlighted. B) Missing values imputation: visualization of missing value replacements. Figure S5: Station 5 NO2 concentration: A)Missing values distribution: The missing regions are highlighted. B) Missing values imputation: visualization of missing value replacements. Figure S6: Station 6 NO2 concentration: A)Missing values distribution: The missing regions are highlighted. B) Missing values imputation: visualization of missing value replacements. Figure S7: Station 7 NO2 concentration: A)Missing values distribution: The missing regions are highlighted. B) Missing values imputation: visualization of missing value replacements. Figure S8: Station 8 NO2 concentration: A)Missing values distribution: The missing regions are highlighted. B) Missing values imputation: visualization of missing value replacements. Figure S9: Station 9 NO2 concentration: A)Missing values distribution: The missing regions are highlighted. B) Missing values imputation: visualization of missing value replacements. Figure S10: Station 10 NO2 concentration: A)Missing values distribution: The missing regions are highlighted. B) Missing values imputation: visualization of missing value replacements. Figure S11: Station 11 NO2 concentration: A)Missing values distribution: The mi [file 40537_2023_754_MOESM1_ESM.pdf]

RESEARCH

Artificial Intelligence for improving Nitrogen Dioxide forecasting of Abu Dhabi environment agency ground-based stations

Additional information

Table S1: Missing Value Percentages in the 20 UAE monitoring stations

Station	Missing Value (Percentage)
Station 1	4.52%
Station 2	3.68%
Station 3	6.80%
Station 4	7.19%
Station 5	7.31%
Station 6	5.83%
Station 7	6.15%
Station 8	1.51%
Station 9	1.56%
Station 10	5.80%
Station 11	1.05%
Station 12	2.45%
Station 13	62.64%
Station 14	5.98%
Station 15	1.17%
Station 16	3.17%
Station 17	2.52%
Station 18	5.75%
Station 19	6.32%
Station 20	11.15%

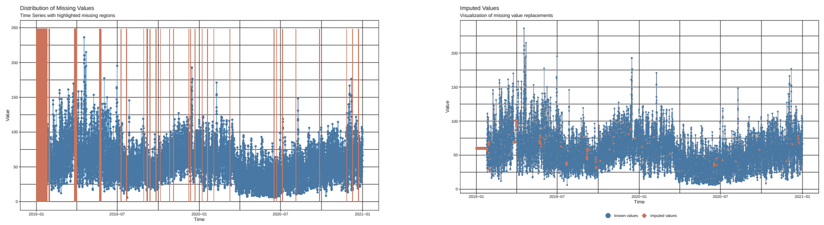

(a) Missing values distribution (b) Missing values imputation

Figure S1: **Station 1 NO_2 concentration:** A)Missing values distribution: The missing regions are highlighted. B) Missing values imputation: visualization of missing value replacements

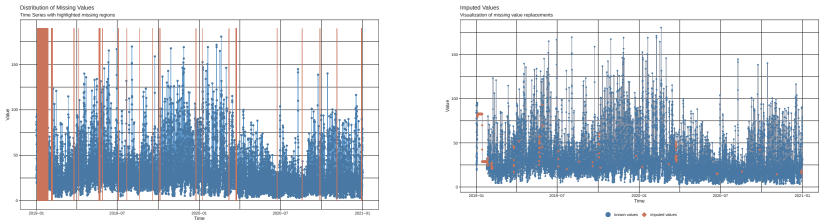

(a) Missing values distribution (b) Missing values imputation

Figure S2: **Station 2 NO_2 concentration:** A)Missing values distribution: The missing regions are highlighted. B) Missing values imputation: visualization of missing value replacements

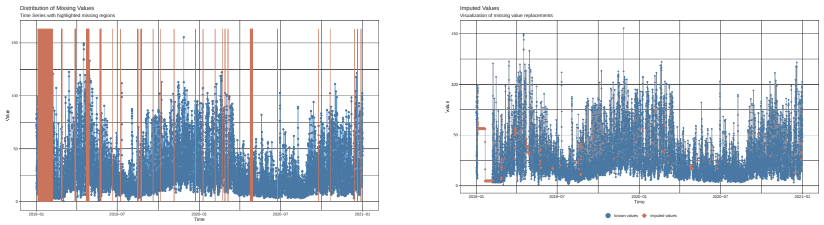

(a) Missing values distribution (b) Missing values imputation

Figure S3: **Station 3 NO_2 concentration:** A)Missing values distribution: The missing regions are highlighted. B) Missing values imputation: visualization of missing value replacements

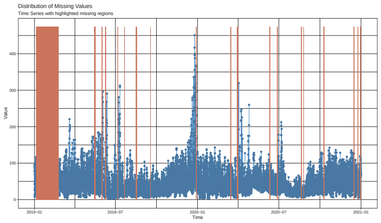

(a) Missing values distribution

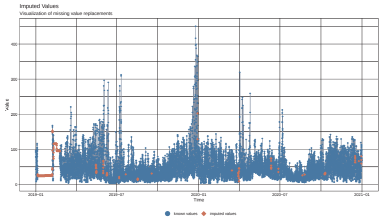

(b) Missing values imputation

Figure S4: **Station 4 NO_2 concentration:** A)Missing values distribution: The missing regions are highlighted. B) Missing values imputation: visualization of missing value replacements

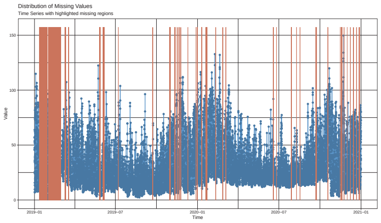

(a) Missing values distribution

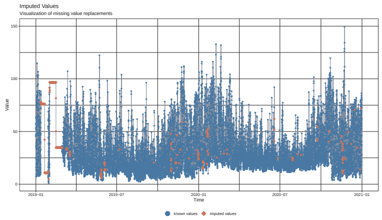

(b) Missing values imputation

Figure S5: **Station 5 NO_2 concentration:** A)Missing values distribution: The missing regions are highlighted. B) Missing values imputation: visualization of missing value replacements

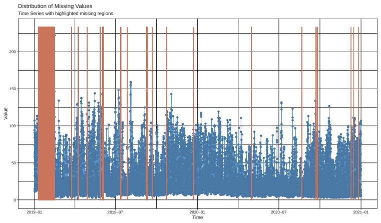

(a) Missing values distribution

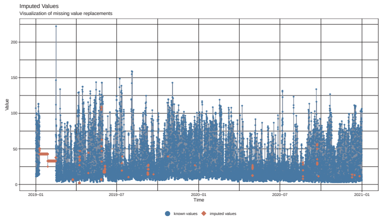

(b) Missing values imputation

Figure S6: **Station 6 NO_2 concentration:** A)Missing values distribution: The missing regions are highlighted. B) Missing values imputation: visualization of missing value replacements

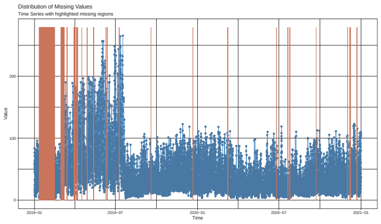

(a) Missing values distribution

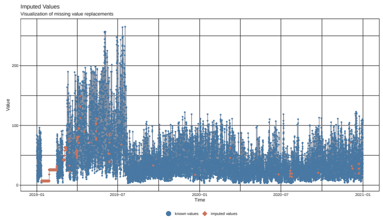

(b) Missing values imputation

Figure S7: **Station 7 NO_2 concentration:** A)Missing values distribution: The missing regions are highlighted. B) Missing values imputation: visualization of missing value replacements

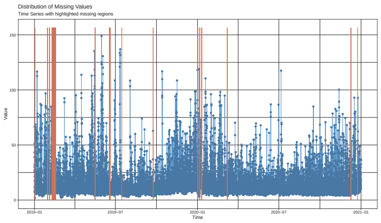

(a) Missing values distribution

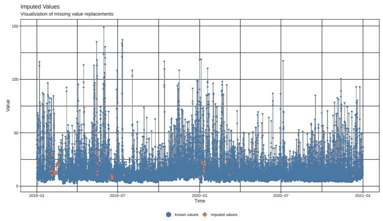

(b) Missing values imputation

Figure S8: **Station 8 NO_2 concentration:** A)Missing values distribution: The missing regions are highlighted. B) Missing values imputation: visualization of missing value replacements

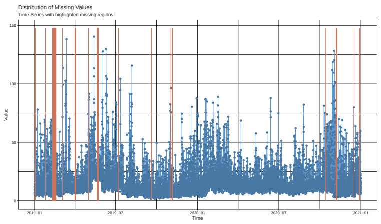

(a) Missing values distribution

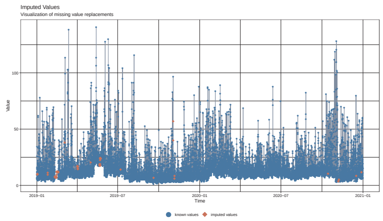

(b) Missing values imputation

Figure S9: **Station 9 NO_2 concentration:** A)Missing values distribution: The missing regions are highlighted. B) Missing values imputation: visualization of missing value replacements

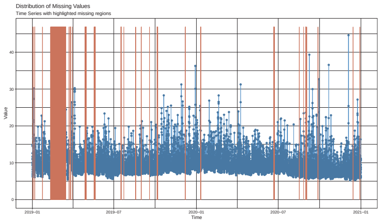

(a) Missing values distribution

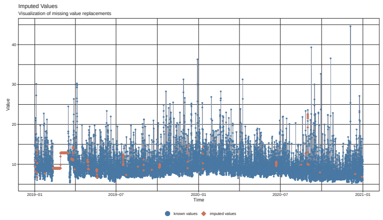

(b) Missing values imputation

Figure S10: **Station 10 NO_2 concentration:** A)Missing values distribution: The missing regions are highlighted. B) Missing values imputation: visualization of missing value replacements

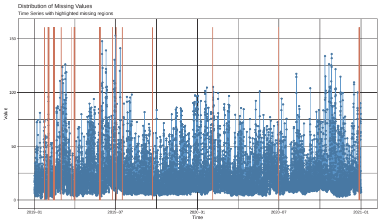

(a) Missing values distribution

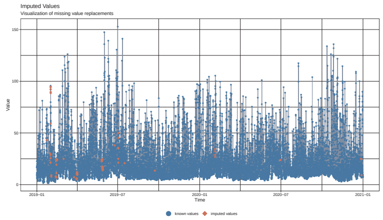

(b) Missing values imputation

Figure S11: **Station 11 NO_2 concentration:** A)Missing values distribution: The missing regions are highlighted. B) Missing values imputation: visualization of missing value replacements

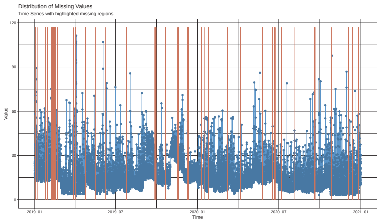

(a) Missing values distribution

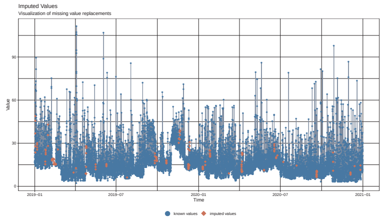

(b) Missing values imputation

Figure S12: **Station 12 NO_2 concentration:** A)Missing values distribution: The missing regions are highlighted. B) Missing values imputation: visualization of missing value replacements

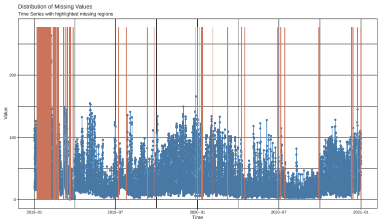

(a) Missing values distribution

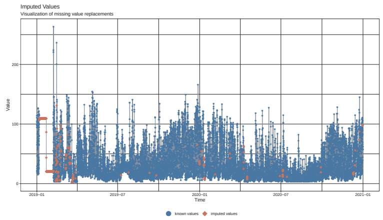

(b) Missing values imputation

Figure S13: **Station 14 NO_2 concentration:** A)Missing values distribution: The missing regions are highlighted. B) Missing values imputation: visualization of missing value replacements

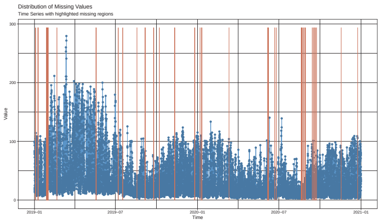

(a) Missing values distribution

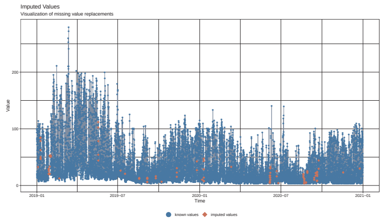

(b) Missing values imputation

Figure S14: **Station 15 NO_2 concentration:** A)Missing values distribution: The missing regions are highlighted. B) Missing values imputation: visualization of missing value replacements

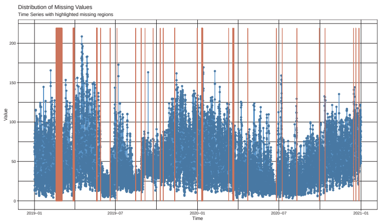

(a) Missing values distribution

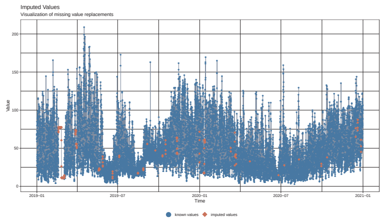

(b) Missing values imputation

Figure S15: **Station 16 NO_2 concentration:** A)Missing values distribution: The missing regions are highlighted. B) Missing values imputation: visualization of missing value replacements

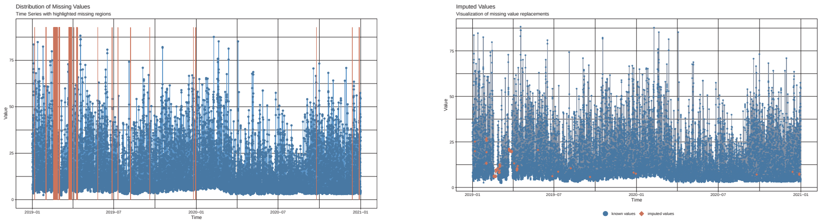

(a) Missing values distribution (b) Missing values imputation

Figure S16: **Station 17 NO_2 concentration:** A)Missing values distribution: The missing regions are highlighted. B) Missing values imputation: visualization of missing value replacements

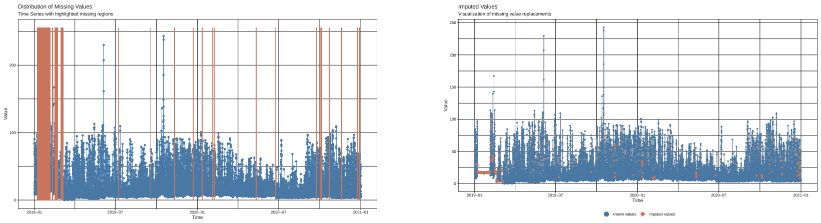

(a) Missing values distribution (b) Missing values imputation

Figure S17: **Station 18 NO_2 concentration:** A)Missing values distribution: The missing regions are highlighted. B) Missing values imputation: visualization of missing value replacements

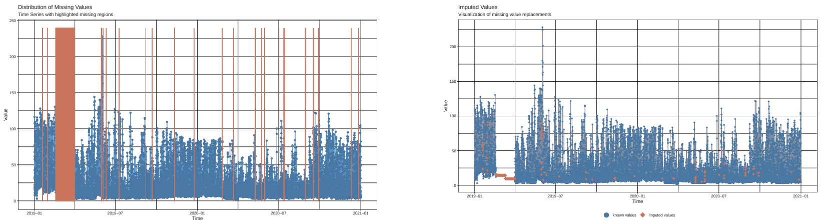

(a) Missing values distribution (b) Missing values imputation

Figure S18: **Station 19 NO_2 concentration:** A)Missing values distribution: The missing regions are highlighted. B) Missing values imputation: visualization of missing value replacements

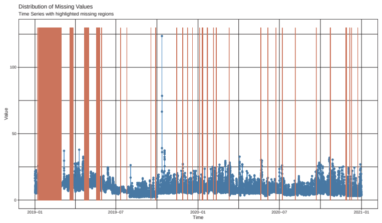

(a) Missing values distribution

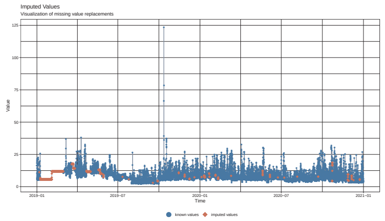

(b) Missing values imputation

Figure S19: **Station 20 NO_2 concentration:** A)Missing values distribution: The missing regions are highlighted. B) Missing values imputation: visualization of missing value replacements
